# “A Tree Must Be Bent While It Is Young”: Teaching Urological Surgical Techniques to Schoolchildren

**DOI:** 10.5812/numonthly.1637

**Published:** 2012-03-01

**Authors:** Stefan Buntrock

**Affiliations:** 1 Klinik am Kurpark, Center for Rehabilitative Urology, Bad Wildungen, Germany

**Keywords:** Urology, Child, Teaching

## Abstract

**Background:**

Playing video games in childhood may help achieve advanced laparoscopic skills later in life. The virtual operating room will soon become a reality, as “doctor games 2.0” will doubtlessly begin to incorporate virtual laparoscopic techniques.

**Objectives:**

To teach surgical skills to schoolchildren in order to attract them to urology as a professional choice later in life.

**Materials and Methods:**

As part of EAU Urology Week 2010, 108 school children aged 15–19 attended a seminar with lectures and simulators (laparoscopy, TUR, cystoscopy, and suture sets) at the 62nd Congress of the German Society of Urology in Düsseldorf. A Pub-Med and Google Scholar search was also performed in order to review the beneficial effects of early virtual surgical training. MeSh terms used were “video games,” “children,” and “surgical skills.” Searches were performed without restriction for a certain period of time.

**Results:**

In terms of publicity for urology, EAU Urology Week, and the German Society of Urology, the event was immensely successful. Regarding the literature search, four relevant publications were found involving children. An additional three articles evaluated the usefulness of video gaming in medical students and residents.

**Conclusions:**

Making use of virtual reality to attract and educate a new generation of urologists is an important step in designing the future of urology.

## 1.Background

Each year in September, the European Association of Urology (EAU) encourages urological societies in Europe to create public awareness about urological diseases. This can be done through small local events with lectures in a small community hospital or through a large, centrally organized meeting that may draw media attention. As an example, the 2008 Urology Week in oslo had numerous lectures and an enormous walk-through prostate model, and was broadcasted to approximately 3 million Norwegians through television and radio. All urologists are invited to participate in Urology Week, and more information can be found on www.urologyweek.org. Since its introduction in 2008, approximately 20 countries regularly take part in EAU Urology Week, informing people about prostate cancer, urinary incontinence, and erectile dysfunction. In 2010, the German Society of Urology (DGU) took the event a step further by presenting urology to possible future colleagues—schoolchildren aged 15 to 19. Under the motto “Become a urologist for one day,” they were invited to attend a special session at the DGU Congress in Düsseldorf including hands-on training in surgical techniques.

owing to the rapid progress in the development of minimally invasive techniques, urology has changed. however, even more so than today, future urologists will depend on well-developed eye-hand coordination along with advanced spatial skills. To provide training on these skills during childhood, it has been suggested that playing video games may have positive effects on laparoscopic performance later in life. owing to major advances in virtual reality (VR), video games simulating laparoscopic surgery are not only widely accessible for aspiring young urologists but are also on the verge of entering childhood bedrooms as “doctor games 2.0” for video consoles. The technical aspects of surgery require well-developed psychomotor abilities that should be learned as early as possible. Furthermore, it has been shown that video games enhance spatial skills in children (1). Video game experts with high-volume training over long time periods outperformed non-gamers in a number of basic cognitive skills, and the differences remained even after intensive training sessions for the non-gamers (2). These findings underline the possible benefits of video gaming at a young age, despite a possible selection bias reported by the authors of the latter study.

## 2. Objectives

our initiative basically aimed to encourage the future generation of doctors to develop their basic skills in laparoscopy as early as possible, preferably with a career in urology in mind. This was because hardly any undergraduates entering medical school consider urology as their first career choice. The prejudiced prospect of a profession connected to urine and older men prevents many aspiring doctors from considering urology. Moreover, undergraduate urological education is rudimentary in quite a few European countries, with a quarter of all European medical students not receiving any formal training (3).

## 3. Materials and Methods

Recruitment was carried out through active communication with schools in the wider Düsseldorf area and by issuing several press releases prior to the event. Every 15-year-old and above considering a career in medicine was eligible to participate. The course was a one-day class at the venue of the congress and was held on two separate days to maximize the number of participants. The program started with two lectures that introduced aspects of urology. Then, small groups of 5–6 participants moved for approximately two hours between different workstations, where they were introduced to a variety of practical urological tasks. There were five stations: laparoscopy, transurethral resection (TUR), cystoscopy, ultrasound, and tying knots/suturing. The equipment used was made available by Karl Storz Gmbh, hitachi Medical Systems Gmbh, and B. Braun Melsungen AG. TUR was the only station that used a VR environment. Authentic equipment was used to simulate laparoscopy and cystoscopy. To make this exercise more enjoyable, we used jelly bears as training objects that had to be removed out of groups of red peppers. For suturing, bananas and bacon were used. Residents from the German Society of Residents in Urology (GESRU) oversaw each group. The session concluded with a lecture on science and the purpose of scientific congresses.

In order to evaluate the usefulness of childhood spatial and psychomotor training, a literature search through PubMed and Google Scholar was performed, using the MeSh terms “video games,” “children,” and “surgical skills.”

## 4. Results

### 4.1. “Become a Urologist for One Day”

A total of 108 schoolchildren, aged 15 to 19, attended the seminar. Media attention was extensive, with three major TV stations and two regional and national newspapers covering the event. Feedback given by the participants themselves was very positive.

### 4.2. Results and Discussion of the Literature Review

#### 4.2.1. Video Games and VR-Laparoscopic Performance in Children

A relatively short time after video games became widely accessible, a study showed that they could improve spatial skills in young girls and boys (1). The best results were seen in children starting out with relatively poor spatial skills. Furthermore, the gender differences that were measured initially became less pronounced. however, video games have changed fundamentally since then. Marble Madness(1994), used in the previous study, cannot be compared to today’s three-dimensional action games. In addition to spatial skills, modern action games can often enhance attention capabilities in gamers versus non-gamers, likely because they require divided attention regarding visual impressions, attention in time, space, and objects (4, 5). The effect of early, regular gaming on advanced laparoscopic performance later in life was scrutinized by van Dongen et al. (6). They compared medical interns (mean age 24 years) to schoolchildren (mean age 12.5 years) using the lapSim® virtual reality laparoscopic simulator. Interns with experience in video games significantly outperformed all other groups at four basic endoscopic skill tasks. Interestingly, schoolchildren with video game experience played on average 8.6 hours per week, whereas interns with experience played only 1.9 hours. however, experienced children scored as well as inexperienced adults in terms of total scores, efficiency, and speed. The authors concluded that the benefits of early gaming might be blurred by “the difference in psychomotor development between the ages of 12 and 24 years.” however, they do not discuss performance in relation to the accumulated total number of training hours. For the learning curve it will make a difference whether one has played extensively for two years or for twelve years. A similar study was carried out by Rosenthal et al. (7). In contrast to the former study, board-certified surgeons were included as an additional group. Furthermore, differences in psychomotor skill levels were controlled for by validated conventional tests. Not surprisingly, board-certified surgeons showed the best VR performance, whereas children with little prior exposure to video games scored lowest. however, after correction for multiple testing, the differences between children with high and those with low experience and between residents and certified surgeons no longer reached statistical significance. This could be attributed to the shape of the learning curve, where differences become less pronounced once the steep part of the curve is overcome. While residents had a comparable economy of movement and similar precision, board-certified surgeons were much faster. The findings from this study support the notion that video games enhance spatial and surgical performance in both children and adults. Additionally, it showed that neither medical nor surgical background is needed to carry out basic tasks in laparoscopic surgery.

#### 4.2.2. Video Games and VR-Laparoscopic Performance in Adults

Residents in surgical specialties and medical students have been studied quite frequently in terms of a correlation between video game experience and laparoscopic skills. Boyle et al.(2012) looked at physical and virtual laparoscopic performance in medical students with no experience in laparoscopy, no experience with the Nintendo Wii™ and no periods of regular video gaming (8). In this randomized controlled trial, two groups were built who had to perform two physical and one virtual laparoscopic simulated task at baseline and seven days later. one group served as the control group and did not train during those seven days. The other group was trained on the Nintendo Wii™. Although statistically not significant, this latter group reached a higher level of proficiency. As a possible reason for the lack of significance, the authors speculated about an insufficient training schedule with too few practice hours and large time gaps. In a large study of 279 medical students without prior experience in VR laparoscopy (VRl), video game expertise was a predictive factor of higher baseline VRl scores through multivariate analysis (9). Interestingly, factors related to self-confidence were also predictive of first time VRl performance. These included “self-confident to assist in a laparoscopic operation (after the VRl session), video game playing, interest in surgery, intended surgical residency, and self-estimation of fine motor skills.” Rosser et al. found in residents that past video game experience, verified by demonstrated video game skill, highly correlated with laparoscopic skill and suturing ability (10).

#### 4.2.3. Video Games and Laparoscopic Performance in the Operating Room

There has been little interest in the question of whether video gaming enhances operating room performance in established surgeons. however, it has been shown that a preoperative warm-up video game session significantly enhances laparoscopic surgery performance on basic tasks in novices (11). Despite striking similarities between VRl and real laparoscopy, VRl has one major disadvantage: its virtual nature. There is no tactile feedback from the tissues, which is an important aspect of laparoscopy (12). This might explain the findings from a trial that included certified gynecologic surgeons (13). The authors found a negative correlation between increasing operating room experience/age and simulator performance. obviously, VRl has its limitations, as there are important aspects of laparoscopy that for the time being cannot be simulated well enough.

## 5. Discussion

Playing video games (especially 3D action games) on a regular basis trains cognitive skills essential for laparoscopic surgery. It is quite possible that medical games that incorporate aspects of laparoscopy will be put on the market. It is clear that schoolchildren thinking about a career in medicine stand only to gain from playing video games frequently, in terms of spatial, sensorimotor, and attention skills. Introducing the PlayStation generation to urology at an early age (similar to our initiative “Become a urologist for one day”) is likely to bring new talent to the specialty within a few years. however, it remains somewhat unclear whether today’s computer-literate children, who will eventually become surgeons, will actually outperform all others. Future research should focus more on this topic.
